# Pediatric Collagenous Gastroenteritis and Colitis Presenting as Protein-Losing Enteropathy

**DOI:** 10.14309/crj.0000000000001028

**Published:** 2023-04-10

**Authors:** Kailey A. Remien, Marisa Mancuso, Kevin Watson

**Affiliations:** 1 Department of Pediatric Gastroenterology, Akron Children's Hospital, Akron, OH

**Keywords:** pediatrics, gastroenterology, colitis, collagenous colitis, diarrhea

## Abstract

There is a lack of literature on pediatric collagenous colitis. This is a report of a child with collagenous gastroenteritis and colitis who presented with chronic, nonbloody diarrhea and lower extremity edema secondary to protein-losing enteropathy. Collagenous colitis is rare in children; collagenous gastroenteritis and colitis are even less documented; and this diagnosis does not typically present with protein-losing enteropathy. The pediatric patient in this report had a presentation of a rare disease. Her disease self-resolved, and she has remained asymptomatic without pharmacologic intervention. This illness should be considered in a child presenting with this constellation of symptoms.

## INTRODUCTION

Collagenous colitis is a type of microscopic inflammation of the large intestine, which typically occurs in middle-aged patients. It is an uncommon diagnosis in pediatrics yielding a low index of suspicion, which can delay diagnosis.^1^ Collagenous gastroenteritis with colonic involvement, as seen in the presented patient, has an even lower prevalence.^2^ There is also no standardized treatment strategy.^2^ This patient's collagenous gastroenteritis presented as protein-losing enteropathy, which is common in children, but less common in the setting of collagenous gastroenteritis. The differential diagnosis for protein-losing enteropathy includes causes of hypoalbuminemia, such as malabsorption, abnormal synthesis, nephrotic syndrome, burns, inflammation of the vasculature, and hemodilution, and causes of mucosal injury, such as infection, inflammatory bowel disease, lymphoma, celiac disease, vasculitic disorders, lymphatic abnormalities, and obstruction.^3^

## CASE REPORT

A 3-year-old White girl presented to the emergency department for 2 weeks of diarrhea. Stools were liquid, occurring 3 times daily. She had poor oral intake and decreased urination. The family denied fever, congestion, black or bloody stools, or changes in diet. The patient had no significant medical history, no recent medication treatment or travel, and no family history of gastrointestinal disease. The family also denied increased juice intake and felt it was not associated with lactose intake. The patient was well-appearing, well-hydrated, and in no acute distress. Abdominal examination was unremarkable; x-ray showed a nonobstructive gas pattern without stool burden. Urine analysis had small ketones. The patient was able to tolerate fluid intake and was discharged home with education on the importance of oral hydration.

The patient continued to have diarrhea over the next few days and developed fatigue and anuria. She was then admitted. The patient had nonpitting edema of the lower extremities bilaterally to the distal shins. A complete metabolic panel was only remarkable for a hypoalbuminemia of 2.0 g/dL (3.2–4.5 g/dL). Gastrointestinal and respiratory multiplex polymerase chain reaction assays were negative for detectable infections. Urine protein was negative, and again urine had small ketones. Chest x-ray; complete blood count; C-reactive protein; and immunoglobulin (Ig) A, IgM, IgG, and transglutaminase IgA were within normal limits. Owing to no history of frequent infections and normal Ig levels, common variable immunodeficiency was less likely. Postinfectious protein-losing enteropathy was the leading diagnosis, despite no confirmed infection. After 1 day of dextrose 5% in normal saline, the patient's energy returned as her edema improved. She was discharged home with scheduled follow-up.

Over the following month, the patient continued to have diarrhea and lost 1.9 kg. Stools were brown, large, and explosive, occurring 2–5 times per day. The mom had cut juice from the patient's diet without improvement and tried to give probiotics, but the patient refused. No other dietary changes were made. She was referred to pediatric gastroenterology. Workup included a complete blood count, complete metabolic panel, Igs, thyroid stimulating hormone, ceruloplasmin, iron level, lipid panel, endomysial antibody, deaminated gliadin, stool alpha-1-antitrypsin, stool pH, stool-reducing substances, fecal fat, stool calprotectin, and stool occult blood. Remarkable results include a triglyceride level of 834 mg/dL (0–74 mg/dL), ceruloplasmin at 11.5 mg/dL (21.7–43.3 mg/dL), low IgG at 151 mg/dL (453–916 mg/dL), elevated IgA at 108 mg/dL (20–100 mg/dL), stool alpha-1-antitrypsin elevated at 64 mg/dL (≤54 mg/dL), and calprotectin elevated at 329 μg/g (≤50 μg/g). Abdominal ultrasound showed no abnormalities. Echocardiogram and EKG were unremarkable.

**Figure 1.**
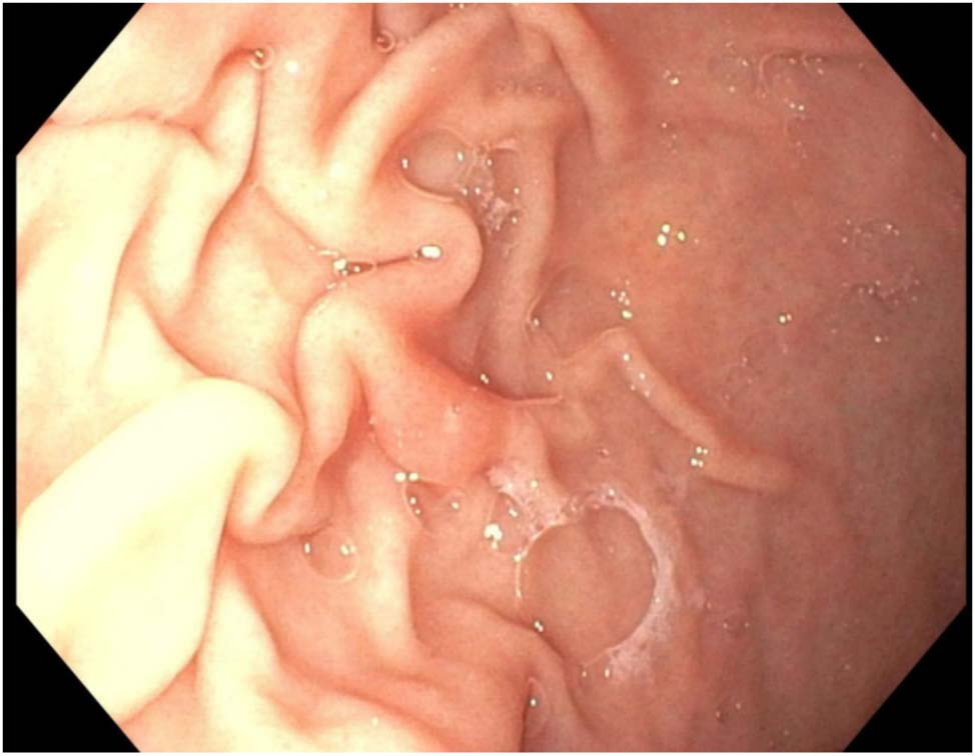
Gross visualization of the stomach mucosa during upper endoscopy.

**Figure 2.**
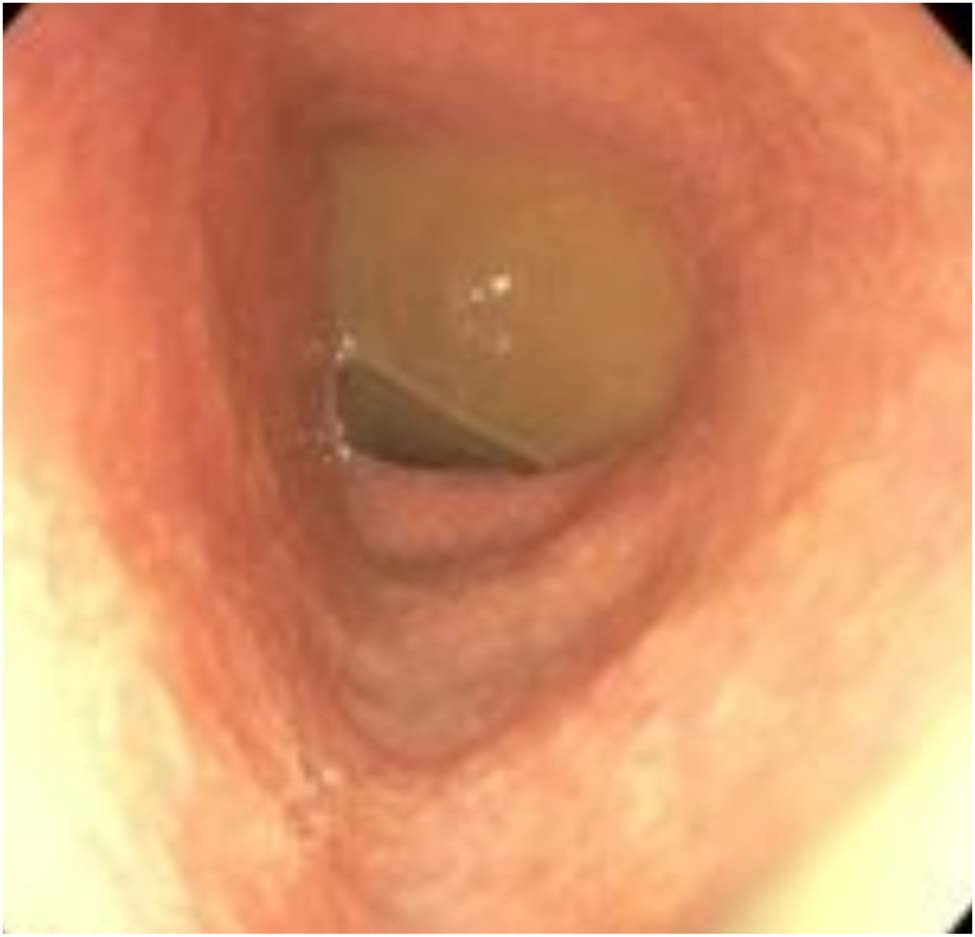
Gross visualization of the colonic mucosa during colonoscopy.

**Figure 3.**
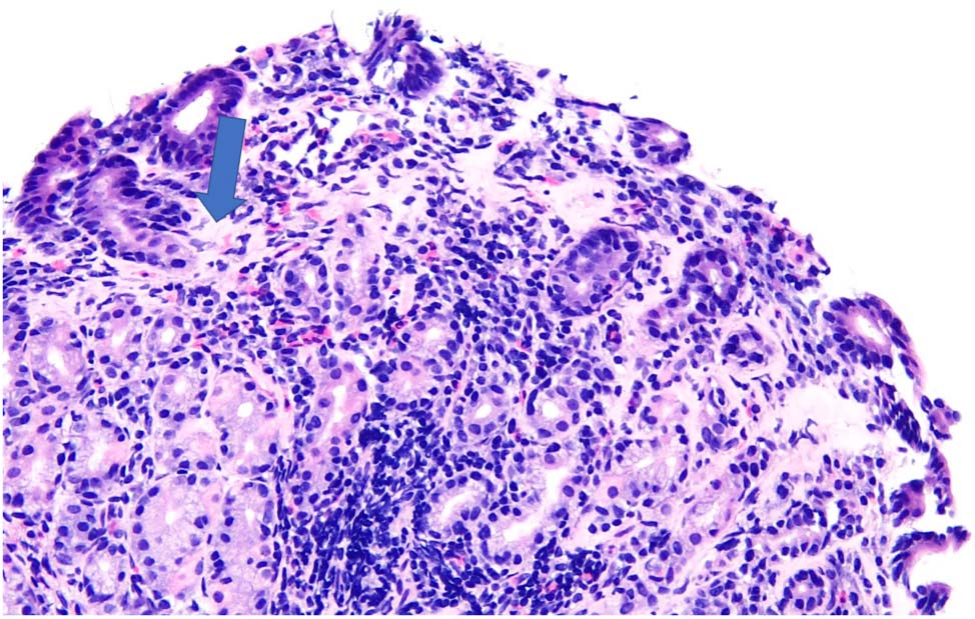
Stomach antrum biopsies ×20, antral mucosa with mild chronic gastritis, and equivocal prominence in lamina propria eosinophils. Focal thickening of the subepithelial collagen table. The blue arrow is pointing to the collagen deposition (pink area) found on biopsy.

**Figure 4.**
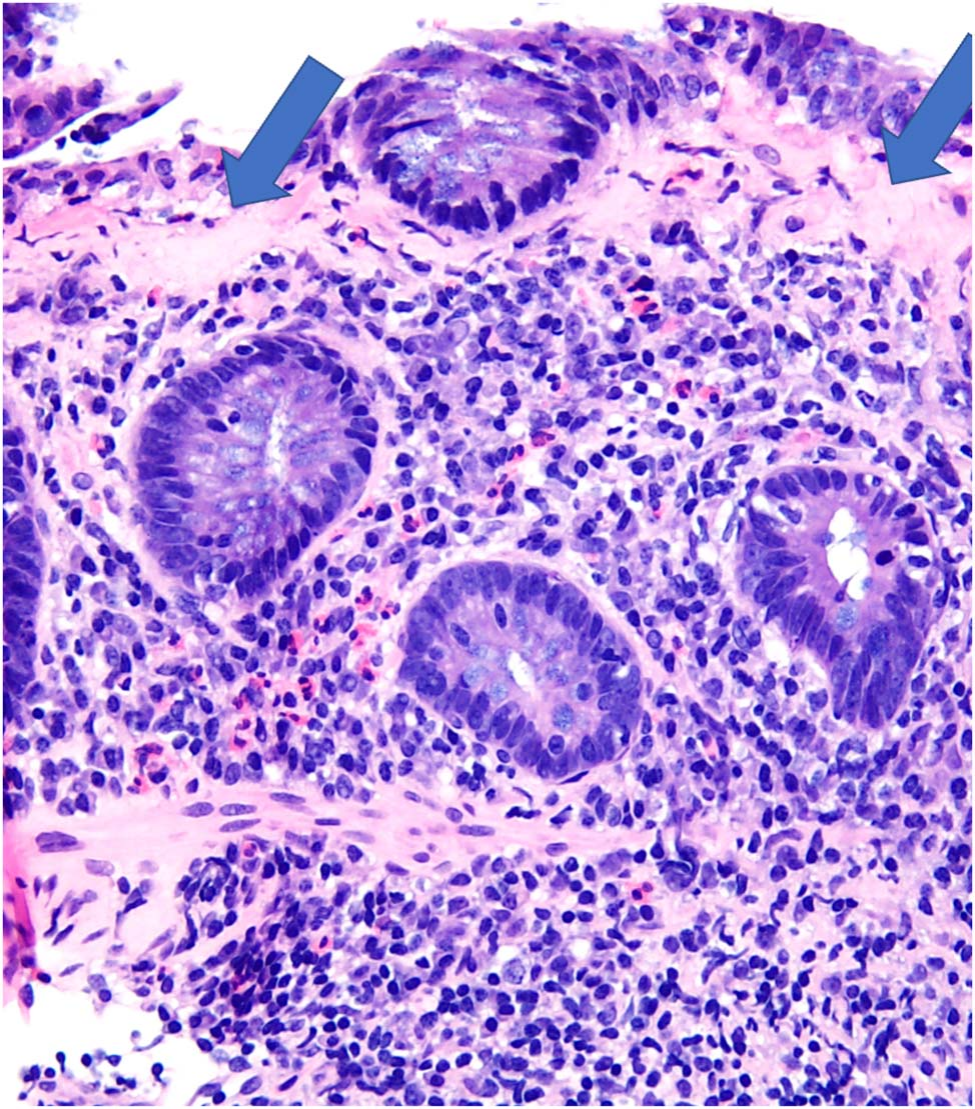
Left **c**olon biopsy ×20, colonic mucosa with collagenous colitis pattern; increased subepithelial collagen table and intraepithelial lymphocytes. The blue arrows are pointing to the collagen deposition (pink area) found on biopsy.

Endoscopy showed normal esophageal and gastric mucosa (Figure 1) with mild congestion of the duodenal mucosa with blunting of the villi. There was unspecified colitis with diffuse erythema from the rectum to the cecum with distal congested mucosa (Figure 2). Biopsies showed chronic gastritis with prominence of eosinophils in the lamina propria and thickening of the subepithelial collagen table (Figure 3), severe villous blunting in the duodenum and terminal ileum with thickening of the subepithelial collagen table, and collagenous colitis of the left colon (Figure 4). MOC31, BerEP4, and CD10 stains of duodenal tissue were normal. Electron microscopy of duodenal tissue had no evidence of microvillus abnormalities or other pathology. Microscopic review showed no signs of autoimmune enteropathy.

A diagnosis of collagenous gastroenteritis and colitis was made. Gastroenterology recommended treatment with budesonide for 8 weeks. The patient took this medication for 1 week before refusal. Diarrhea had lessened in severity, occurring once daily, and stools began to be firmer.

Owing to improvement, the family did not force medication. Diarrhea resolved within a few weeks. Repeat laboratory tests demonstrated normalization of albumin, triglycerides, and Igs.

## DISCUSSION

Collagenous colitis is characterized by chronic, nonbloody diarrhea, and colicky abdominal pain.^4^ Colonoscopy biopsies demonstrate increased inflammation including an increase in the number of lamina propria plasma cells and possibly eosinophils along with prominent lymphocytes. This will be in combination with subepithelial collagenous thickening.^5^ Many adults with collagenous colitis have a concurrent autoimmune disease.^7^ In children, juvenile scleroderma, hypothyroidism, diabetes mellitus, *Aeromonas hydrophilia* infection, and other autoimmune disorders are associated with collagenous colitis and gastritis.^6,7^

There is only 1 recent report of a child who presented with a protein-losing enteropathy found to have collagenous colitis and only 2 adult cases.^1^ Owing to low prevalence, the index of suspicion for collagenous colitis in pediatric patients remains low. Collagenous colitis should be on the differential for patients with persistent diarrhea of unknown etiology. It should also be considered in the differential for protein-losing enteropathy.

Treatment with corticosteroids, specifically budesonide, is supported by the literature and this patient. One pediatric study showed that 9 of 17 patients with collagenous colitis were treated with corticosteroids. Five were treated with oral prednisone with an average dose of 0.55 mg/kg/d with a mean duration of 4.7 months. Four patients were treated with budesonide for an average of 3.7 months. Eight of the 9 reached full or partial remission within 8 weeks.^8^

This is a report of a child with collagenous gastroenteritis and colitis who presented with chronic, nonbloody diarrhea and lower extremity edema secondary to a protein-losing enteropathy.

Collagenous colitis is rare in children, with a combination of collagenous gastroenteritis and colitis being less documented. This pediatric patient had a presentation of a rare disease.

## DISCLOSURES

Author contributions: KA Remien and M. Mancuso drafted the initial manuscript. K. Watson critically reviewed and revised the manuscript. All authors approved the final manuscript as submitted and agree to be accountable for all aspects of the work. K. Watson is the article guarantor and accepts full responsibility for the conduct of this study.

Financial disclosure: None to report.

Informed consent was obtained for this case report.

Previous presentation: The case report was previously presented as a poster presentation at the North American Society for Pediatric Gastroenterology, Hepatology and Nutrition annual conference; October 13, 2022; Orlando, Florida.
